# Family and clinical indicators of domestic violence among pregnant women in Ilorin, North-Central Nigeria

**DOI:** 10.11604/pamj.2022.41.1.29893

**Published:** 2022-01-03

**Authors:** Adedayo Yemi Kofoworade, Kola Moradeyo Alabi, Louis Okeibunor Odeigah, Ampitan Amoko

**Affiliations:** 1 Department of Family Medicine, University of Ilorin Teaching Hospital, Ilorin, Kwara State, Nigeria,; 2 Tobi Hospital, Off Taiwo Road, Ilorin, Kwara State, Nigeria

**Keywords:** Domestic violence, family functioning, anaemia, pregnant women

## Abstract

**Introduction:**

domestic violence affects one in four families and has significant health consequences on sufferers. When it occurs among pregnant women, it can be associated with pregnancy-related complications. There is dearth of data on the association between certain family and clinical factors of pregnant women, and domestic violence in Nigeria. This study was conducted to determine the prevalence and pattern of domestic violence and its association with certain clinical factors and family functioning of pregnant women attending the antenatal clinic at the University of Ilorin Teaching Hospital, Ilorin, North-Central Nigeria.

**Methods:**

a total of 333 respondents were recruited for the cross-sectional study between June and August, 2017 using systematic sampling technique. Structured questionnaires were used to obtain information about domestic violence and family functioning among the study participants. Blood pressure, urinalysis and packed cell volume of respondents were obtained following standard procedures. Data were analyzed using SPSS-20 and Chi-square was used to identify significant risk factors for domestic violence among the study subjects.

**Results:**

the results of this study showed that the prevalence of domestic violence among the study population was 34.5%. The most common form of violence in this study was psychological aggression (74.8%), followed by sexual coercion (47.8%), then physical assault (14.8%) and physical assault with injury (3.5%). There was a statistically significant association between domestic violence and; 1) family dysfunction p<0.001); 2) anaemia (p<0.001).

**Conclusion:**

pregnant women presenting with anaemia as well as those from dysfunctional families are particularly at high risk of domestic violence and as such, special attention should be drawn to these factors with a view to screen and identify victims of abuse during routine antenatal clinic visits.

## Introduction

Domestic Violence (DV) is a pattern of abusive behaviours by one or both partners in an intimate relationship such as marriage, dating, cohabitation or within the family [1]. Domestic violence, known as the most common type of gender-related violence, affects nearly one in three women globally and has significant health consequences [2]. It is an important problem because it is global, violates fundamental human rights of women and is a major public health problem [3]. Forms of domestic violence include but not limited to physical assault, sexual abuse and psychological violence [4]. Researchers have described domestic violence during pregnancy as a global public health problem due to its adverse health consequences and intervention potential [5,6]. The subject has also become popular among policy makers and some international human right movement groups who are interested in women´s health. It is known that pregnancy neither provides immunity from domestic violence nor reduces the risk of abusive relationship. Some researchers have even argued that pregnancy constitutes a period of heightened risk for domestic violence irrespective of the woman´s age, race, socioeconomic status or educational level [7,8]. Pregnant women are at a higher risk of experiencing gender-based violence because they are more likely to be in relationships compared to non-pregnant women. In addition, their age (15-49 years old) has also been identified a higher risk group for domestic violence [9]. Also, wrong opinions about pregnancy, abnormal emotions of the partner regarding pregnancy, and reduction in sexual contact are some other factors that make pregnant women vulnerable [10].

Violence can affect pregnancy through direct and indirect mechanisms [11]. A blow to a pregnant woman´s abdomen can cause adverse outcome directly. The indirect mechanisms are related to a woman´s victimization experience from domestic violence and how it can induce intermediate risks (such as psychological stress) that could cause poor pregnancy outcomes like foetal injury, placenta abruption among others. James and colleagues, in a Meta-analytic review of studies from selected developed and developing countries, found a wide variation in the prevalence of domestic violence among pregnant women, with figures ranging from 4.8-63.4% [12]. A similar and more recent systematic review in Nigeria found a range of between 2.3% and 44.6% [9]. This variation is probably attributable to differences across studies in the sampled population, as well as differences in definitions and methodologies. There are evidences that the overall prevalence of domestic violence during pregnancy in less developed countries is higher than that in developed countries [5,12,13]. In Nigeria, Onon and colleagues in Southeast Nigeria found an overall prevalence of 44.6% in the index pregnancy with verbal abuse being the most common form (60.1%), whereas emotional and physical abuse constituted 21% of the reported forms of domestic violence [14]. In a similar study in Northern Nigeria, the overall prevalence was 7.4%, the majority reported being physically assaulted (58.6%) [15]. Gyuse and Ushie, in another study among pregnant women in Jos, North central Nigeria, found that 63.5% had experienced domestic violence. Of these, 26.5% were physically abused, 38.0% had endured verbal insults, whereas sexual and emotional insults accounted for 10.7% and 1.4%, respectively.

The family unit which is seen as a medium of socialization has been reported to be a place where much violence is directed at its female members [16]. One potential influencer of domestic violence that has received very little attention is family functioning. Family functioning is defined as the way in which family members interact, react to, and treat other family members; It includes variables within the family such as communication, styles, tradition, clear roles and boundaries, and the degree of fusion, flexibility, adaptation and resilience [17]. Dysfunction in a family occurs when there is a conflict, misbehaviour on the part of individual family members continually, leading other members to accommodate such actions [18]. Family dysfunction has an important influence and provides powerful source of information about the risk for future violence [19]. Studies have also reported that abusive families have poorer family functioning, more problems and more problematic relationships [20,21]. Also, families where there is domestic violence have poor family relationships and provide low levels of support and protection [22]. On the other hand, there is evidence that women who had adequate family support were less likely to be abused by their husbands [23].

Furthermore, the relationships between domestic violence and some obstetric complications, like anaemia and hypertensive disorders of pregnancy, have also been studied. Pregnant women who experience domestic violence have been found to have a higher risk obstetric complications, for example hypertension, and anaemia [6,24]. On the other hand however, there was no significant association between domestic violence and hypertensive disorders of pregnancy in a survey of pregnant women in Belgium [25]. Also, Hoang and colleagues in China found no significant association between domestic violence and anaemia among pregnant women in Vietman [26]. While domestic violence may occur in as many as one of every four families [27], studies on family factors associated with development and maintenance of abuse remain scarce all over the world. Also, apart from some of the negative health outcomes of domestic violence in pregnancy, it has also been reported as a contributing factor to maternal mortality [24]. Therefore, there is need to quantify the problem, synthesize information on common risk factors among the parturient population. This study sought to provide evidence about undetected domestic violence, as well as its association with family functioning and certain clinical factors among pregnant women attending antenatal clinics at University of Ilorin Teaching Hospital, Ilorin, Nigeria. Such information can help to advocate for health interventions that will contribute to safe motherhood and healthy babies.

## Methods

**Study area and sampling:** it was a hospital-based descriptive cross-sectional study. The study was conducted at the antenatal clinic of University of Ilorin Teaching Hospital (UITH), Ilorin. Ilorin is the capital city of Kwara State, located in the North-central geopolitical zone of Nigeria. Kwara state shares boundaries with five other states, namely: Kogi, Ekiti, Osun, Oyo and Niger. Ilorin metropolis consists of three local governments areas: Ilorin-South, Ilorin-West and Ilorin-East. University of Ilorin Teaching Hospital is located in Oke-ose, Ilorin-East local government area. It is a 600-bed tertiary health institution. The study participants were all consenting pregnant women, aged 18 years and above attending the antenatal clinic of UITH, Ilorin. However, pregnant women with background low packed cell volume (e.g. those with haemoglobinopathies) and those that were acutely ill, were excluded from the study. The required sample size was determined using Leslie Kish´s Statistical Formula for estimating minimum sample size in health studies [28].


$$n=\frac{Z^{2}pq}{d^{2}}$$

n= desired sample size when population is greater than 10,000 z= standard normal deviation, usually set at 1.96 which corresponds to 95% confidence level. p= proportion of the target population estimated to have a particular characteristic. The prevalence of domestic violence among pregnant women during the index pregnancy in primary care setting in Jos, North central Nigeria of 31.8% [29] was considered for the calculation of sample size. Thus, p= 0.318; q= 1-0.318= 0.682; d= degree of accuracy desired, usually set at 0.05. Thus, n= (1.96) (1.96) (0.318) (0.682) = 333.26; (0.05) (0.05).

An attrition rate of 10% was projected to allow for non-responders and poorly filled questionnaires. Thus, an approximate sample size of 370 was used for the study. A systematic sampling method was used for selection of subjects into the study. Data was collected over a three-month period. The number of participants interviewed every clinic day in order to make a sample size of 370 was 370 (sample size) ÷ 60 (5 working days/week x 4 weeks/month x 3 months) i.e. 370/60, which gave approximately 6. Using the information from the health record department of the hospital, the average daily attendance at the antenatal clinic was 50, then monthly attendance was 50 x 20 (daily attendance x number of working days in a month), giving 1000. Sampling frame was thus 1000 x 3 (monthly clinic attendance x study period in months) which gave 3,000; and sampling interval (sampling frame ÷ sample size) was 3000/370 which gave 8. Therefore, on every clinic day, each folder was assigned a number from 1 to the last. The first subject was selected by simple balloting among the first 8 prospective participants. Thereafter every 8th consenting patient was selected for the study until the required sample size for each day was obtained. The folder of each patient selected was labelled and the hospital number written in a research register to avoid the pitfall of double sampling of the subject. In cases where the ballot picked a subject that had already been included in the sample, the next consenting candidate that met the inclusion criteria was selected. This procedure was repeated every clinic day until the total sample size of 370 was obtained.

**Data collection tools:** structured domestic violence screening tool, the Revised Conflict Tactics Scale-2 (CTS2) was used to determine the prevalence and pattern of domestic violence among the study participants. CTS2 measures a total of 39 behaviours under five categories/scales viz; negotiation, psychological aggression, physical assault, sexual coercion and Injury [30]. The prevalence rate is the percentage of the sample who reported one or more instances of the acts in each category during the index pregnancy. The validity and reliability of CTS2 has been shown to be good and has been used in studies on domestic violence in Nigeria [31,32]. Respondents´ family functioning was assessed by family APGAR questionnaire. This is also a structured questionnaire used to assess a family member´s perception of family functioning by examining his or her satisfaction with family relationship. The measure consists of five parameters of family function: adaptability, partnership, growth, affection and resolve. The response options were designed to describe frequency of feeling satisfied with each parameter on a 3-point scale ranging from 0 (hardly ever) to 2 (almost always). The correlation of the instrument with the previously validated instrument (Pless-Satherwhite index) is 0.80 and correlation with clinical report is 0.64 [33]. Family APGAR has been validated and used for a previous study in Nigeria [18]. Family APGAR´s scores are interpreted as follows: 7-10 = highly functional family; 4-6 = moderately dysfunctional family; 0-3= severely dysfunctional family.

Based on the history and urinalysis results, respondents with hypertensive disorders of pregnancy was classified into four groups as recommended by the National High Blood Pressure Education Program Working Group on High Blood Pressure in Pregnancy: 1) chronic hypertension, 2) preeclampsia-eclampsia, 3) preeclampsia superimposed on chronic hypertension, and 4) gestational hypertension (transient hypertension of pregnancy) (1). Chronic hypertension is high blood pressure that either precedes pregnancy, is diagnosed within the first 20 weeks of pregnancy, or does not resolve by the 12-week postpartum check-up. Gestational hypertension, formerly known as pregnancy-induced hypertension or PIH, is the new onset of hypertension after 20 weeks of gestation. The diagnosis requires that the patient have; elevated blood pressure (systolic ≥ 140 or diastolic ≥ 90 mm Hg, the latter measured using the fifth Korotkoff sound), previously normal blood pressures, no protein in the urine, no manifestations of pre-eclampsia/eclampsia. Preeclampsia is defined as elevated blood pressure after 20 weeks of gestation (≥ 140 mm Hg systolic or ≥ 90 mm Hg diastolic) plus proteinuria (> 0.3 g/24 hours). Eclampsia is the development of convulsions in a pre-existing pre-eclampsia or it may appear unexpectedly in a patient with minimally elevated blood pressure with or without proteinuria [34]. The blood pressure of each patient was measured by auscultatory method using a standard mercury sphygmomanometer with appropriately sized cuff and a lithmann stethoscope. Urinalysis was performed on urine collected by each patient into a clean sample bottle; to look for the presence or absence of protein, using dipstick method (reagent strips). Packed Cell Volume (PCV) was measured from the blood sample collected into the heparinized capillary tube using the haematocrit centrifuge. Participants´ PCV was read with microhaematocrit reader. Respondents were categorized into two groups according to the World Health Organization´s threshold used to define anaemia among pregnant women [35]; those with packed cell volume less than 33% (anaemic group) and those with 33% and above (normal PCV group).

**Data analysis:** the collected data were analyzed using the version 20 software packages of the Statistical Package for Social Sciences (SPSS-20). Results were presented using frequency tables and charts. Frequency distribution was generated to reveal percentages and proportions of the various variables. Associations between independent and dependent variables was tested and assessed. Chi-square was used for categorical variables. The level of significance of this study was set at 5% (p<0.05).

**Ethical consideration:** the approval to undertake the study was obtained from Ethical Review Committee of University of Ilorin Teaching Hospital. Written informed consent was obtained from each participant before being recruited into the study.

## Results

**Figure 1:**
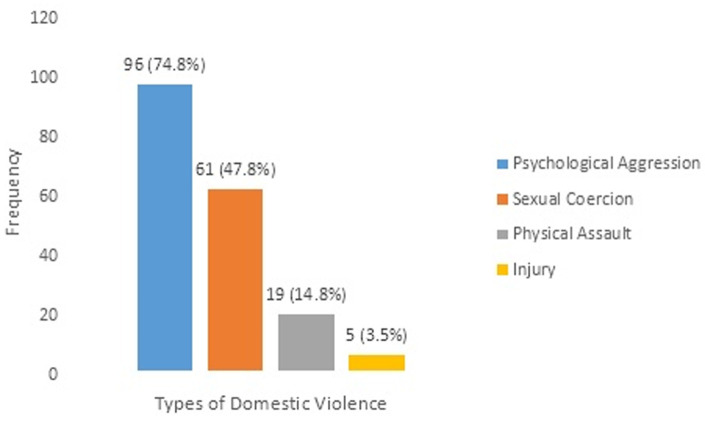
pattern of domestic violence among pregnant women in Ilorin, North-central Nigeria

Table 1 shows the socio-demographic/pregnancy-related characteristics of respondents. A total of three hundred and seventy (370) pregnant women´s data were analyzed. The age range was from 18-46 years, with a mean age of 29.47 ± 4.84 years. Most of the participants were in the age group of 21-30 years (61.1%) and a slight majority were pregnant with their first child (36.5%). Majority of the participants had tertiary education (74.6%) and a high proportion of them wanted the index pregnancy (94%). Also, most of the respondents were married during the study period (96.5%) and were mostly from monogamous families (93.5%). Furthermore, most of the participants were Yorubas (89.2%) and 66.5% practiced Islam. In addition, 0.9% of participants reported occasional alcohol intake. Table 2 shows that, the more dysfunctional the families were, the higher the rate of domestic violence among them (x^2^=58.723, p<0.001). Table 3 shows the relationship between domestic violence and clinical factors of respondents. Respondents, who were anaemic, were more likely to suffer domestic violence than those with normal packed cell volumes and this association was statistically significant (p<0.001, x^2^=45.135). In contrast, the observed trend of domestic violence and presence or absence of hypertensive disorders of pregnancy did not show significant association among the study population (p=0.054, x^2^=9.299). A total of 128 of the 370 participants (34.5%) in this study reported having experienced domestic violence during the index pregnancy (Table 2 and Table 3). The violence type with the highest occurrence in this study was psychological aggression (74.8%), followed by sexual coercion (47.8%), then physical assault (14.8%) and physical assault with injury (3.5%) (Figure 1).

**Table 1:** socio-demographic parameters of respondents (N=370)

Variable	Frequency	Percent
**Age (years)**		
≤ 20	4	1.1
21- 30	226	61.1
31 - 40	134	36.2
> 40	6	1.6
Mean ± SD	29.47 ± 4.84	
Range	18 - 46	
**Number of children**		
None	135	36.5
1	108	29.2
> 1	127	34.3
**Pregnancy intent**		
Wanted	348	94.0
Unwanted	22	6.0
**Religion**		
Christianity	124	33.5
Islam	246	66.5
**Education**		
No formal	19	5.1
Primary	15	4.1
Secondary	60	16.2
Tertiary	276	74.6
**Marital status**		
Currently married	357	96.5
Not currently married	13	3.5
**Type of family (n = 321)**		
Monogamous	346	93.5
Polygamous	24	6.5
**Substance use**		
None	367	99.1
Alcohol	3	0.9
**Ethnicity**		
Hausa	5	1.3
Igbo	21	5.7
Yoruba	330	89.2
Othersa	14	3.8

a Others are the other ethnic groups found in Kwara N=Total number of participants

**Table 2:** association between family functioning and domestic violence

	Domestic violence				
Family functioning	Yes n1(%)	No n2(%)	Total N	χ2	p value
**Highly functional**	73 (25.0)	220 (75.0)	293	58.723	<0.001*
**Moderately dysfunctional**	41 (69.6)	18 (30.4)	59		
**Severely dysfunctional**	14 (76.9)	4 (23.1)	18		

**χ2:** Chi square test, *: p value < 0.05 (statistically significant) **n1 and n2:** number of participants in each category in which DV is present and absent respectively

**Table 3:** association between domestic violence and the clinical factors

	Domestic violence				
Variable	Yes	No	Total	χ2	p value
	n1 (%)	n2 (%)	N		
**Anaemia**					
Anaemic	79 (68.7)	71 (32.4)	150	40.106	< 0.001*
Not Anaemic	36 (31.3)	148 (67.6)	184		
**Hypertension**					
No Hypertension	90 (78.3)	190 (86.8)	280	8.524Y	0.074
Chronic hypertension	2 (1.7)	10 (4.6)	12		
Preeclampsia	13 (11.3)	10 (4.6)	23		
Chronic hypertension with preeclampsia	4 (3.5)	1 (0.5)	5		
Gestational hypertension	6 (5.2)	8 (3.7)	14		

**χ2:** Chi square test**, Y:** Yates corrected, *: p value < 0.05 (statistically significant) **n1=** number of participants with domestic violence in which clinical factors are present or absent **n2=** number of participants without domestic violence in which clinical factors are present or absent

## Discussion

This cross-sectional study showed that 34.5% of the pregnant women in the study area had experienced domestic violence during the index pregnancy. With reference to a study with similar operational definition, this prevalence rate is higher than 5.4% rate earlier reported in Northern Israel [36]. The high prevalence rate observed in this study may be related to the willingness of the index study participants to disclose their experiences of domestic violence and the fact that respondents were interviewed face-to-face by the researcher unlike the Israel study where interview was conducted via telephone conversation. A study done in the US showed that African American women disclosed domestic violence more often in face-to-face than using computer-based technologies [37]. Also, the increasing awareness about domestic violence in recent times could also make participants to report violent behaviours when asked unlike what the situation had been before now. It is however lower than 66.45% rate reported in Iran [38]. The difference could be explained by the socio-cultural and geographical differences between these areas and the fact that the Iran study participants were recruited through a convenience sampling technique. The convenience sampling technique usually leads to a sample that may not be representative of the population and the results may not be reproducible in future studies [39]. The most common form of DV was psychological aggression at 74.8%, followed by sexual coercion (47.8%), then physical assault (14.8%) and physical assault with injury (3.5%). Psychological abuse, as the most common form of violence, was similarly reported by Olagbuji in Benin [40], Umeora in Abakaliki [41] and Hoang in Vietnam [26]. This was expected because psychological abuse usually predates other forms of violence in an abusive relationship. Also, this form of abuse is usually adopted by the perpetrator rather than physical abuse, so as not to inflict harm to the baby in utero. The burden of sexual coercion among this study population is strikingly high (47.8%) although still second to psychological abuse. The finding may also be related to the willingness of the respondents to disclose their experiences of sexual abuse and the fact that respondents were interviewed using the non-judgmental approach in an atmosphere that assured them of confidentiality. In Iran, sexual abuse was the least commonly reported violence among the study subjects [42]. In Jos however, it was the commonest form of domestic violence, occurring in almost two-thirds of the cases [29]. Although, physical assault with or without injury was the least commonly experienced violence act by participants in this study, the proportion of sufferers is nonetheless of concern when compared with a previous study [36]. The direct effect of physical violence on materno-fetal health can lead to pregnancy loss, placenta abruption as well as foetal injury. It suffices to say also, that this might have been underreported which is a common phenomenon with issues of domestic violence [43,44].

There was a statistically significant association between domestic violence, and family functioning of respondents. The more dysfunctional the families were, the higher the rate of domestic violence among them. Pregnancy is always a stressful time, but for women who lack adequate family support systems, it maybe even more difficult. Furthermore, lack of family support may act as a buffer in a couple experiencing a crisis, and the absence of strong structures may cause an increased risk of domestic violence. Although, there is paucity of data on the association between domestic violence and family functioning among pregnant women, this association has been studied among several other populations with consistent findings as the index study. Zheng *et al*. in a cross-sectional study of pregnant women in China found that family dysfunction was a risk factor for DV [45]. In another cross-sectional study of University students in Jordan, family dysfunction was also a significant predictor for perpetration of violent behaviours [19]. Faridand colleagues in Parkistan found that women who had adequate social support were less likely to be abused by their husbands [23]. Among a group of women attending a family medicine clinic in Mexico, higher rate of partner violence was recorded among respondents from dysfunctional families when compared with those from functional families [46]. Also, Singh B and colleagues in Uttar Pradesh, India, found that if a couple makes joint decisions in household matters; there was a 24% reduction in the prevalence of domestic violence [47]. Furthermore, there was a significant association between anaemia and domestic violence in this study. This is similar to what was found in a prospective cohort study by Kaye *et al*. in Uganda who also found that abused pregnant women were more likely to have pregnancy-related complications like anaemia [48]. This could be explained by the fact that pregnant women in abusive relationship are less likely to have autonomy as regards the choice and the quantity of foods they require to sustain themselves before and during pregnancy resulting in nutritional anaemia. Another possible reason may be related to the fact that they are also less likely to be regular on antenatal clinic visits, comply with the routinely prescribed haematinics and other ANC measures that could prevent anaemia. Meanwhile, Hoang and colleagues in China found no significant association between domestic violence and anaemia among pregnant women in Vietman [26].

## Conclusion

This study observed a high prevalence of domestic violence (34.5%) among pregnant women attending the antenatal clinics at the University of Ilorin Teaching Hospital, Ilorin, Kwara State, Nigeria. Psychological aggression was the most common form of abuse (74.8%) while physical assault with injury was the least common (3.5%). Also, respondents from dysfunctional families and those with anaemia were more likely to be victims of domestic violence. Therefore, routine screening for domestic violence during antenatal clinics may be beneficial for early case identification and management. Maternal health care providers should be adequately equipped to offer help to families who are willing to improve their inter-family relationships to achieve healthy family environment and to lower the incidences of violence within homes.

**Recommendation:** clinicians should endeavour to screen pregnant women for domestic violence regularly, especially when there is evidence of family dysfunction and clinical or laboratory evidence of anaemia.

**Limits/strength:** the research instrument (CTS2) used for assessing domestic violence in this study was based on self-report and is thus prone to self-report bias. Respondents could have overestimated or underestimated in their responses. Also, because domestic violence was assessed within the period of the index pregnancy, the ability of the respondents to recall events within this period could have affected the prevalence rate found. However, CTS2 has good sensitivity and specificity when compared with other domestic violence screening tools. Furthermore, this study being a cross sectional hospital-based study means that the various significant associations between the variables tested were not necessarily causal. Only a prospective or randomized controlled study can confirm the causal relationship between these variables. The data generated however, are actionable for clinicians and provide a baseline for future research

### What is known about this topic

- There is evidence that domestic violence is common among pregnant women all over the world and it is associated with poor pregnancy outcomes.

### What this study adds

- Domestic violence may be a contributing factor to the high burden of anaemia among pregnant women in developing countries;
- Dysfunction in a family may be a pointer to an ongoing domestic violence;
- Pregnant women in Ilorin do suffer from physical assault with injuries as a result of domestic violence.
